# The Emerging Role of l-Glutamine in Cardiovascular Health and Disease

**DOI:** 10.3390/nu11092092

**Published:** 2019-09-04

**Authors:** William Durante

**Affiliations:** Department of Medical Pharmacology and Physiology, University of Missouri, Columbia, MO 65212, USA; durantew@health.missouri.edu

**Keywords:** l-glutamine, l-glutamate, ammonia, metabolism, Krebs cycle, cardiovascular disease

## Abstract

Emerging evidence indicates that l-glutamine (Gln) plays a fundamental role in cardiovascular physiology and pathology. By serving as a substrate for the synthesis of DNA, ATP, proteins, and lipids, Gln drives critical processes in vascular cells, including proliferation, migration, apoptosis, senescence, and extracellular matrix deposition. Furthermore, Gln exerts potent antioxidant and anti-inflammatory effects in the circulation by inducing the expression of heme oxygenase-1, heat shock proteins, and glutathione. Gln also promotes cardiovascular health by serving as an l-arginine precursor to optimize nitric oxide synthesis. Importantly, Gln mitigates numerous risk factors for cardiovascular disease, such as hypertension, hyperlipidemia, glucose intolerance, obesity, and diabetes. Many studies demonstrate that Gln supplementation protects against cardiometabolic disease, ischemia-reperfusion injury, sickle cell disease, cardiac injury by inimical stimuli, and may be beneficial in patients with heart failure. However, excessive shunting of Gln to the Krebs cycle can precipitate aberrant angiogenic responses and the development of pulmonary arterial hypertension. In these instances, therapeutic targeting of the enzymes involved in glutaminolysis such as glutaminase-1, Gln synthetase, glutamate dehydrogenase, and amino acid transaminase has shown promise in preclinical models. Future translation studies employing Gln delivery approaches and/or glutaminolysis inhibitors will determine the success of targeting Gln in cardiovascular disease.

## 1. Introduction

Cardiovascular disease is the primary cause of morbidity and mortality in the world, accounting for nearly one-third of all deaths [1]. Aside from its profound effect on the quality and duration of life, cardiovascular disease imposes a severe and costly demand on health services and is expected to surpass the medical cost for all chronic diseases [2]. Although the age-adjusted mortality rate for cardiovascular disease has diminished in industrialized countries owing to life-style changes, smoking cessation, advances in biomedical research, and improvements in medical care and technologies, the aging population and burgeoning epidemic of cardiometabolic disease characterized by obesity, insulin resistance, dyslipidemia, impaired glucose tolerance, and hypertension, threatens to reverse this progress, underscoring the requirement for additional therapeutic options that target this deadly disease.

Substantial evidence indicate that amino acids play a fundamental role in the cardiovascular system. While amino acids serve as basic building blocks for protein synthesis and constitute an important energy source, a select group has been widely studied in the context of cardiovascular disease. Decades of research have established the importance of l-arginine in promoting cardiovascular health through the generation of the gas nitric oxide (NO) by the enzyme NO synthase (NOS) [3,4,5]. The release of NO by endothelial cells (ECs) regulates blood flow and blood pressure by inhibiting arterial tone. Furthermore, NO maintains blood fluidity and prevents thrombosis by limiting platelet aggregation and adhesion. NO also protects against intimal thickening by blocking smooth muscle cell (SMC) proliferation, migration, and collagen synthesis. Moreover, NO mitigates the development of atherosclerosis by blocking the inflammatory response within the vessel wall. Interestingly, l-homoarginine, a derivative of l-arginine, also elicits beneficial effects in the circulation. Clinical studies indicate that low circulating levels of l-homoarginine independently predicts mortality from cardiovascular disease while high levels are associated with reduced mortality. The mechanism mediating the protection by l-homoarginine is not known but likely involves its capacity to stimulate NO formation by serving as a substrate for NOS. Contrarily, extensive work has identified l-homocysteine, a sulfur containing amino acid formed from the metabolism of l-methionine, as an independent risk factor for atherosclerosis [6]. The atherogenic action of l-homocysteine has been attributed, in part, to its ability to impair the bioavailability of NO. Studies in the past decade have also revealed the complex and contradictory actions of l-tryptophan and its myriad of metabolites in regulating cardiovascular function [7]. Finally, although the role of l-glutamine (Gln) in nutrition and health have been extensively documented, its effects on the cardiovascular system have just recently come to light [8,9,10,11]. In this review, we describe the metabolism and function of Gln in cardiovascular physiology and pathology and highlight potential therapeutic approaches that target this amino acid in cardiovascular disease. 

## 2. l-Glutamine Metabolism

**Figure 1:**
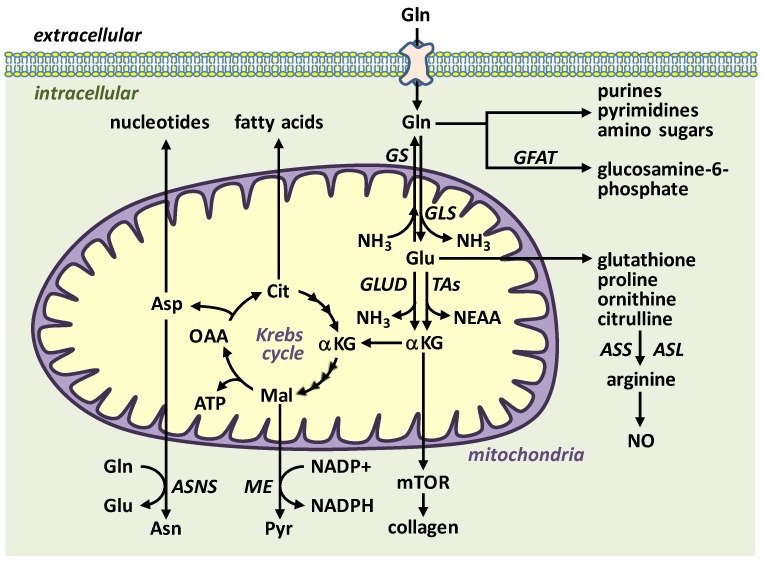
Overview of l-glutamine (Gln) transport and metabolism. Gln is transported into cells by various transporters and preferentially metabolized to l-glutamate (Glu) and ammonia (NH_3_) by the mitochondrial enzyme glutaminase (GLS). In contrast, the enzyme glutamine synthetase (GS) condenses NH_3_ to Glu to form Gln while the enzyme glutamine:fructose-6-phosphate amidotransferase (GFAT) transfers Gln’s amino group to fructose-6-phosphate to generate glucosamine-6-phosphate. Gln and Glu can be converted to a number of important molecules, including amino acids, fatty acids, nucleotides, adenosine triphosphate (ATP), glutathione, and Krebs cycle intermediates. Asn, asparagine; ASNS, asparagine synthetase; Asp, aspartate; ASS, argininosuccinate synthetase; ASL, argininosuccinate lyase; Cit, citrate; GLUD, glutamate dehydrogenase; αKG, α-ketoglutarate; Mal, malate; ME, malic enzyme; mTOR, mammalian target of rapamycin; NEAA, nonessential amino acids; NO, nitric oxide; OAA, oxaloacetate; Pyr, pyruvate; TAs, transaminases.

Gln is the most abundant and versatile amino acid in the body and plays a critical role in nitrogen exchange between organs, intermediary metabolism, immunity, and pH homeostasis [9,10,11]. This nutrient is classified as a conditionally essential amino acid, as endogenous synthesis may be insufficient to meet optimal demands under conditions of catabolic stress, critical illness, and in preterm infants. Gln is an important substrate for the synthesis of peptides, proteins, lipids, purines, pyrimidines, amino sugars, nicotinamide adenine dinucleotide phosphate (NADPH), glucosamine, antioxidants, and for many other biosynthetic pathways involved in regulating cell function (Figure 1). Several enzymes are involved in Gln metabolism. Gln is predominantly synthesized from l-glutamate (Glu) and ammonia (NH_3_) by the action of the largely cytosolic enzyme Gln synthetase (GS), whereas the mitochondrial enzyme glutaminase (GLS) is responsible for the hydrolysis of Gln to Glu and NH_3_. GS is highly expressed in skeletal muscle, while GLS is found in most cells with the small intestine, kidney, leukocytes, and vascular endothelium possessing the highest activity. There are two distinct isoforms of GLS, GLS1 and GLS2, but GLS1 is the major isoform expressed in cardiovascular tissues [12,13,14]. Gln is also metabolized by glutamine:fructose-6-phosphate amidotransferase (GFAT), which condenses Gln’s amino group and fructose-6-phosphate into glucosamine-6-phosphate, a precursor for N- and O-linked glycosylation reactions [10].

The GLS product Glu is used for the synthesis of the antioxidant glutathione: a small three amino acid peptide (Glu-Cys-Gly) which is an efficient neutralizer of peroxide-based free radicals. Alternatively, Glu is further metabolized by Glu dehydrogenase and/or aminotransferases to α-ketoglutarate, which then enters the Krebs cycle generating ATP and serving as an anaplerotic source of carbon for the formation of non-essential amino acids and lipids. In addition, the production of NADPH by malate-pyruvate cycling promotes redox homeostasis by providing the reducing equivalents for glutathione reductase to regenerate glutathione. Finally, in the intestine, enterocytes convert Glu to delta1-pyrroline-5-carboxylate enabling the formation of l-proline, l-ornithine, and l-citrulline. By generating l-citrulline, which is subsequently metabolized to the NOS substrate l-arginine by the concerted action of argininosuccinate synthetase and argininosuccinate lyase in the kidney, Gln also functions as an l-arginine precursor to drive NO synthesis [15]. 

## 3. l-Glutamine and Cardiometabolic Disease

Cardiometabolic disease is a constellation of metabolic dysfunctions characterized by insulin resistance, impaired glucose tolerance, dyslipidemia, hypertension, and central adiposity. The prevalence of this disease is high and increasing, currently impacting approximately 25% of the adult population in the United States [16]. Individuals with cardiometabolic disease are prone to develop diabetes and future cardiovascular events. Indeed, patients with cardiometabolic disease are two times more likely to die from coronary heart disease and three times more likely to have heart attacks and stroke [17,18]. The pathogenesis of this complex disease is not known but may be related to disturbances in Gln metabolism. A seminal report by Cheng et al. [19] investigated possible pathways underlying cardiometabolic disease by high-throughput metabolic profiling of two large, well-characterized, community-based cohorts (the Framingham Heart Study and the Malmo Diet and Cancer Study). They found that plasma Gln or the Gln:Glu ratio is inversely associated with body mass index, blood pressure, circulating triglycerides and insulin, and positively associated with high density lipoproteins, while plasma Glu levels, alongside branched-chain and other hydrophobic amino acids, are associated with adverse metabolic parameters. Moreover, they describe for the first time that a high Gln:Glu ratio is associated with a lower risk of incident diabetes mellitus, which was subsequently confirmed in a meta-analysis of 46 clinical studies [20]. This study also expanded previous clinical and experimental studies showing that a reduced Gln:Glu ratio is associated with insulin resistant-traits in obese humans and rodents [21,22,23]. A similar inverse association between the serum Gln and established cardiometabolic risk factors is noted in Chinese and Mediterranean populations [24,25,26]. In addition, a recent longitudinal study found that Glu is associated with decreases in insulin secretion and sensitivity, as well as incident type 2 diabetes in a large population of Finnish men [27]. 

Aside from serving as a biomarker for cardiometabolic disease, the Gln-cycling pathway may be an effector of metabolic risk. Acute oral administration of Gln improves glucose tolerance in subjects with or without diabetes, while chronic dietary supplementation of Gln for 6 weeks reduces systolic blood pressure, fasting blood glucose, and improves body composition in patients with type 2 diabetes [28,29]. In addition, Gln administration decreases glycemia in adolescents with type 1 diabetes and in patients undergoing myocardial revascularization [30,31]. Parenteral administration of Gln also prevents the decrease in insulin sensitivity in multiple-trauma patients [32]. Furthermore, Gln, but not Glu, supplementation enhances glucose tolerance and lowers mean arterial blood pressure in mice [19]. Notably, these improvements by Gln are associated with a decline in plasma branched-chain and aromatic amino acids, which have been implicated in insulin-resistance [21]. Beneficial effects of Gln supplementation on some cardiovascular risk factors have also been reported in animals exposed to exercise or a high-fat diet [33,34,35].

A number of mechanisms may mediate the beneficial effects of Gln on cardiometabolic risk, including augmented release of glucagon-like peptide 1, externalization of glucose transporters, stimulation of insulin release by pancreatic β-cells, transcription of insulin-dependent genes, and enhanced insulin disposition [28,32,36]. In addition, the drop in blood pressure by Gln may be due to a rise in NO production secondary to an increase in l-arginine synthesis [15]. Surprisingly, a cross-sectional epidemiological study detected an association of dietary Glu intake with lower blood pressure, but the effect of Glu on endogenous Gln levels was not evaluated [37]. Multiple mechanisms may also underlie the ability of Glu to confer adverse metabolic risk. Glu has been demonstrated to stimulate glucagon release from pancreatic α-cells, which leads to the mobilization of glucose from peripheral tissues to the circulation [38]. In addition, Glu increases the transamination of pyruvate to alanine, a strong promoter of gluconeogenesis that is elevated in obesity [21]. Furthermore, Glu is converted to α-ketoglutarate, an established anabolic agent [39]. Intriguingly, blocking Glu production by targeting hepatic GLS2 activity ameliorates hyperglycemia in both humans and mice, suggesting a possible new therapeutic avenue to treat hyperglycemia. Moreover, inhibiting α-ketoglutarate formation by deleting hepatic Glu dehydrogenase activity blocks gluconeogenesis in mice [40], further highlighting the importance of glutaminolysis in glucose homeostasis.

Finally, a prospective study of older patients with high cardiovascular risk found that baseline levels of circulating Glu are related to increased cardiovascular events (non-fatal stroke, non-fatal myocardial infarction, or cardiovascular death) while the plasma Gln:Glu ratio is related to decreased risk, especially with regard to stroke [41]. In addition, a genome-wide association study identified a genetic variant associated with diminished GS expression, which may lead to a Gln deficiency, to the development of coronary artery disease in type 2 diabetes [42]. Interestingly, small clinical trials indicate that Gln supplementation enhances myocardial repair in patients with coronary artery disease and limits cardiac damage after coronary revascularization [43,44,45]. Moreover, a prospective study in two large, well-defined and independent cohorts showed that dietary intake of Gln and the plasma Gln:Glu ratio were inversely related to the risk of cardiovascular mortality, independent of other dietary or lifestyle factors [46]. Together, these studies illustrate a key protective role for Gln against cardiovascular disease. 

## 4. l-Glutamine and Endothelial Cell Function

The vascular endothelium forms the inner layer of blood vessels and is a crucial regulator of blood vessel structure and function. ECs dynamically modulate vascular permeability, arterial tone, the proliferation and migration of SMCs, thrombosis, and the recruitment and infiltration of leukocytes into the vessel wall by releasing a panoply of mediators, with NO being of paramount importance [47]. The dysfunction or loss of ECs is a well-established response to cardiovascular risk factors that precedes the development of atherosclerosis and other cardiovascular disorders. Recent work by our laboratory and others has established a central role for Gln in promoting EC function [48,49,50]. We found that human EC proliferation and DNA synthesis are absent, and migration significantly curtailed in cells cultured in Gln-free media. Similarly, inhibiting GLS1 expression or activity blocks the proliferation and migration of ECs. This is observed in ECs derived from various species and vascular sources, and likely represents a generalized response to GLS1. We also determined that GLS1 stimulates EC proliferation and motility by fueling the Krebs cycle, which fulfills the necessary energetic and macromolecular requirements of growing and moving cells. Furthermore, the selective loss of GLS1 in ECs mitigates their proliferative response in vivo and results in impaired vessel sprouting in animal models of angiogenesis, supporting a pivotal role for this enzyme in blood vessel formation [49,50]. Interestingly, loss of endothelial GS expression or pharmacological blockade of GS also retards EC migration, but not proliferation, and angiogenesis in pathological mouse models, while only minimally affecting healthy adult quiescent ECs. However, these actions of GS are independent of Gln synthesis and are mediated by the palmitoylation of ras homolog family member J [51]. Thus, GS may represent a disease-restricted target for the therapeutic treatment of angiogenesis in various pathological states. 

The metabolism of Gln by GLS1 also prevents EC senescence, while Gln plays a critical role in EC redox homeostasis by generating glutathione [48,49,50,51,52]. In addition, glutaminolysis is essential for energy production and ion transport in human corneal endothelium [53]. Gln also protects ECs against various harmful stimuli, including oxidative stress, hypertonicity, infection, and hyperglycemia [54,55,56,57]. In contrast, Glu induces oxidative stress and apoptosis in ECs [58]. Furthermore, physiologic levels of Gln reduces the expression of adhesion molecules and migration of leukocytes in ECs activated by preeclamptic plasma or arsenic [59,60]. Moreover, Gln supplementation enhances endothelial progenitor cell mobilization in diabetic and septic mice [61,62], and promotes endothelium-dependent dilation in humans and mice [63,64]. The latter findings are somewhat surprising given that several reports show that Gln inhibits the production of NO by cultured ECs [65,66]. This inhibitory effect is mediated by the metabolism of Gln by GFAT to glucosamine, which inhibits pentose cycle activity and reduces NADPH concentrations that are required for NOS activity [67]. However, administration of Gln to freshly isolated blood vessels increases NO synthesis. These discordant findings may reflect the much lower activity of GFAT in freshly isolated compared to cultured ECs, which may be insufficient to compromise NADPH levels [68]. Additionally, the in vivo administration of Gln may drive NO production by delivering additional substrate (l-arginine) to NOS [15]. 

**Figure 2:**
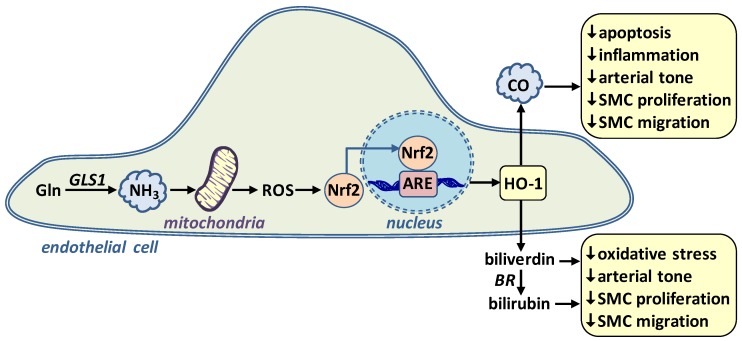
Role of l-glutamine (Gln)-derived ammonia (NH_3_) in stimulating endothelial cell heme oxygenase-1 (HO-1) gene expression and maintaining vascular homeostasis. Gln is metabolized by glutaminase-1 (GLS1) to form the gas NH_3_. NH_3_ stimulates the production of mitochondrial reactive oxygen species (ROS) which causes the activation and translocation of NF-E2-related factor-2 transcription factor (Nrf2) into the nucleus, where it binds to the antioxidant responsive element (ARE) in the promoter region of the gene to trigger HO-1 transcription. HO-1 catalyzes the conversion of heme to carbon monoxide (CO) and biliverdin, the latter being rapidly metabolized to bilirubin by biliverdin reductase (BR). CO and the bile pigments (biliverdin and bilirubin) promote vascular homeostasis by inhibiting apoptosis, oxidative stress, inflammation, arterial tone, and vascular smooth muscle cell (SMC) proliferation and migration.

ECs possess abundant GLS1 activity resulting in high rates of NH_3_ synthesis [48,69,70]. Although long considered a potentially toxic product of GLS1, we recently identified NH_3_ as a novel signaling gas in the vasculature that promotes EC survival [71]. Exogenously administered or Gln-derived NH_3_ stimulates the expression of the enzyme heme oxygenase-1 (HO-1) in human ECs (Figure 2). In addition, dietary supplementation of NH_3_ induces the expression of HO-1 in murine blood vessels. HO-1 is an important enzyme that degrades heme into equimolar amounts of carbon monoxide (CO), iron, and biliverdin [72,73,74]. Biliverdin is promptly metabolized to bilirubin by biliverdin reductase. Both bile pigments (biliverdin and bilirubin) are potent scavengers of reactive oxygen species while CO directly dilates blood vessels and inhibits vascular cell apoptosis. CO and the bile pigments also exert anti-inflammatory effects and block the proliferation and migration of vascular SMCs [75,76]. 

The induction of HO-1 gene transcription by NH_3_ occurs through the activation of the NF-E2-related factor-2 transcription factor by mitochondrial reactive oxygen species. Moreover, we discovered that NH_3_ protects against cytokine-mediated EC death via the HO-1 mediated production of CO. These findings establish NH_3_ as a novel regulator of EC survival and identify a unique signaling pathway by which Gln preserves vascular health. Consistent with these findings, NH_3_ also protects against the lethal actions of tumor necrosis factor-α in renal epithelial cells and improves survival of Gln-starved hybridoma cells, indicating that the cytoprotective action of NH_3_ extends beyond ECs [77,78]. Moreover, by inducing the expression of HO-1, NH_3_ may also inhibit arterial tone, oxidative stress, inflammation, and the activation of vascular SMCs. 

## 5. l-Glutamine and Pulmonary Arterial Hypertension

Pulmonary arterial hypertension (PAH) is a progressive, often fatal condition that is predominantly driven by the hyperproliferation and migration of vascular cells that results in the formation of lumen-obliterative pulmonary vascular lesions [79,80]. This aberrant vascular remodeling response that is accompanied by fibrosis and vasoconstriction leads to increased pulmonary arterial pressure and ultimately to right ventricular cardiac failure and premature death. PAH is triggered by exogenous injuries, such as hypoxia, infections, drugs, and toxins, as well as congenital heart disease and a growing number of mutations, including those in the bone morphogenic protein receptor 2 (BMPR2) gene.

While metabolic reprogramming and mitochondrial dysfunction are known to contribute to the cellular hallmarks of PAH, the importance of Gln metabolism is just beginning to emerge. Extracellular matrix stiffness, which is an early pathologic event in PAH, stimulates the proliferation of pulmonary ECs and SMCs due to the induction of GLS1 by the two transcriptional co-activators Yes Associate Protein 1 (YAP) and Transcriptional Coactivator with PDZ-Binding Motif (TAZ) [12]. GLS1 expression is increased in pulmonary arterioles of the rat monocrotaline model of PAH, and Gln measured in isolated pulmonary ECs is decreased, suggestive of anaplerotic flux through the Krebs cycle. The increase in glutaminolysis in PAH also promotes fibrosis by stimulating collagen translation and stability via the α-ketoglutarate-mediated mammalian target of rapamycin activation and proline hydroxylation [81], sparking a vicious cycle of arterial stiffening, glutaminolysis, and hyperproliferation. Pharmacological inhibition GLS1 activity disrupts this cycle and reduces arterial remodeling and PAH in monocrotaline-treated rats. A similar increase in GLS1 expression or glutaminolysis is observed in rhesus macaques with simian-immunodeficiency virus-associated PAH or in lung samples from patients with human-immunodeficiency virus-mediated PAH. The rewiring of Gln metabolism may also contribute to the maladaptive cardiac remodeling response in PAH, as increases in glutaminolysis have been detected in the right ventricle of both monocrotaline-treated rats and human PAH patients [82].

More recently, PAH patients with abnormal BMPR2 function were found to display a substantial decline in Gln across the transplumonary gradient compared with control subjects, suggesting that mutations in these receptors may impact Gln metabolism [83]. Indeed, ECs or transgenic mice harboring PAH-causing mutations exhibit substantially more Gln-derived carbon throughout the Krebs cycle than wild-type controls. Moreover, BMPR2 mutant ECs display a hyperproliferative phenotype but are completely intolerant of Gln-limiting conditions. Mechanistically, this Gln-addiction is propelled downstream from BMPR2 by oxidant injury of the mitochondria leading to the formation of isoketals which inactivate sirtuin-3 and stabilize hypoxia inducible factor-1α. Moreover, scavenging isoketals normalizes Gln metabolism and prevents PAH in BMPR2 mutant mice. Thus, therapeutic targeting of Gln metabolism represents a promising new approach in treating various forms of PAH. 

## 6. l-Glutamine and other Cardiovascular Disorders

Numerous studies demonstrate that Gln affords protection against ischemia-reperfusion injury. Dietary Gln supplementation in rodents ameliorates ischemia-reperfusion injury in multiple organs, including the small intestine, brain, liver, skeletal muscle, kidney, and heart [84,85,86,87,88,89,90]. Gln elicits many beneficial effects in the setting of ischemia-reperfusion. It reduces oxidative and nitrosative stress and minimizes inflammation by blocking inflammatory mediator release, adhesion molecule expression, the recruitment and infiltration of immune cells into the organ and increases the percentage of alternatively activated macrophages (M2) that support the resolution of inflammation [86,87,89,91]. In addition, Gln inhibits lipid peroxidation, necrosis, and apoptosis following ischemia-reperfusion [85,88,92,93]. The molecular mechanisms by which Gln preserves organ viability and function are varied involving the induction of HO-1 and heat shock proteins and the maintenance of glutathione levels [84,85,88,94,95,96].

Reduced concentrations of plasma and erythrocyte Gln have been reported in patients with sickle cell disease [97]. The low erythrocyte Gln levels in these patients are associated with an altered redox environment that may compromise cell integrity [97,98]. In addition, the Gln:Glu ratio in erythrocytes is inversely correlated with the severity of pulmonary hypertension in patients with sickle cell disease, a complication linked to hemolytic rate. Importantly, oral administration of Gln in patients improves the redox status of sickle red blood cells and reduces their adhesion to human ECs [98]. Moreover, a phase 3 clinical trial found that oral intake of Gln reduces the median number of pain crises in patients with sickle cell disease compared to those who received placebo [99]. Based on this trial, the United States Food and Drug Administration approved pharmaceutical grade Gln as a prescription drug to reduce the rate of acute complications of sickle cell disease. Finally, Gln may also afford protection against myocardial injury. Oral administration of Gln in animals protects against cardiac injury by antineoplastic agents, severe burn damage, and diabetes, in part, by limiting oxidative stress through maintenance of cardiac glutathione metabolism [100,101,102,103]. In addition, Gln supplementation may be beneficial in patients with heart failure [104,105]. 

## 7. Therapeutic Targeting of l-Glutamine in Cardiovascular Disease

**Figure 3:**
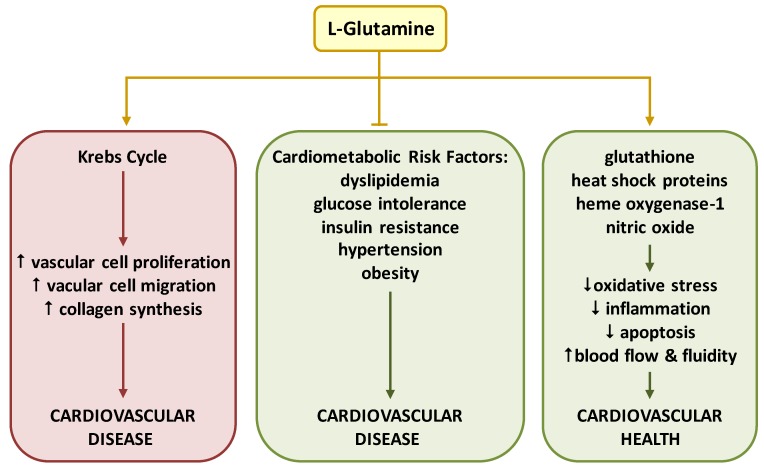
Emerging role of l-glutamine (Gln) in cardiovascular health and disease. Multiple mechanisms mediate the beneficial actions of Gln. In particular, Gln exerts potent antioxidant, anti-inflammatory, and anti-apoptotic effects in the circulation by stimulating the expression of glutathione, heat shock proteins, and heme oxygenase-1. In addition, l-glutamine stimulates blood flow and fluidity by generating nitric oxide. Moreover, Gln alleviates many known risk factors for cardiovascular disease, including dyslipidemia, glucose intolerance, insulin resistance, hypertension, and obesity. However, in some instances, excessive shunting of l-glutamine into the Krebs cycle can promote cardiovascular disease by stimulating aberrant vascular cell proliferation, migration, and collagen synthesis.

There is a growing appreciation for the role of Gln in the cardiovascular system. Gln is a key substrate for the synthesis of DNA, ATP, proteins, and lipids, that drive critical processes in vascular cells, including proliferation, migration, apoptosis, senescence, and extracellular matrix deposition. Moreover, emerging data indicates that Gln elicits both beneficial and detrimental effects on cardiovascular health (Figure 3). Several mechanisms underlie the salutary actions of Gln. In particular, Gln and its metabolites exert potent antioxidant, anti-inflammatory, and anti-apoptotic effects in the circulation by stimulating the induction of HO-1 and heat shock proteins, and the production of glutathione. By serving as an l-arginine precursor, Gln may also preserve vascular homeostasis by improving blood flow and blood fluidity via the synthesis of NO. Importantly, Gln alleviates many known risk factors for cardiovascular disease, such as dyslipidemia, glucose intolerance, insulin resistance, hypertension, and obesity. However, the rewiring of Gln metabolism can also promote the development of cardiovascular disease by fueling the Krebs cycle and stimulating the unrestrained growth and migration of vascular cells and the deposition of extracellular matrix.

Clinical and experimental studies have identified deficiencies in circulating levels of Gln in cardiometabolic disease, hemolytic disorders, and other conditions of stress [9,10,11]. In these instances, dietary supplementation of Gln has proven effective and is now prescribed in patients with sickle cell disease. Gln is usually administered using its free form but more stable dipeptide forms, consisting of l-glycyl-l-glutamine, l-arginyl-l-glutamine, and l-alanyl-l-glutamine have also been utilized. Pharmacokinetic studies indicate that 50–75% of orally administered Gln is extracted by the splanchnic bed in healthy humans [106,107,108]. Despite this extensive extraction, enteral administration of Gln is effective in raising blood levels of Gln in a dose-related manner. Studies in animals and humans have demonstrated increases in circulating Gln between 30 and 120 min following oral supplementation of free Gln or l-alanyl-glutamine [106,107,108,109,110]. Interestingly, the bioavailability of l-alanyl-glutamine may be superior to that of free Gln, and this may reflect the expression of the high capacity human oligopeptide transporter 1 in luminal microvilli of enterocytes which facilitates the uptake of the dipeptide [111]. The use of l-arginyl-l-glutamine is particularly attractive in cardiometabolic and sickle cell disease as deficiencies in both Gln and l-arginine have been detected [112,113,114]. While a multitude of studies have documented the safety of Gln supplementation, caution must be exercised when using this amino acid in critically ill patients [115,116]. As an alternative approach, specific metabolites of Gln with known beneficial effects may be administered rather than the parent molecule. In this regard, the judicious application of NH_3_ should be considered given its ability to stimulate HO-1 gene expression and EC survival, as well as the salutary effects on the cardiovascular system noted with the delivery of other potentially noxious gases, including CO and hydrogen sulfide [117,118,119,120]. 

In some cases, the excessive shunting of Gln to the Krebs cycle may foster cardiovascular disease. Enhanced glutaminolysis contributes to vascular cell proliferation, migration, and collagen synthesis leading to the development of aberrant angiogenic responses and PAH. Several therapeutic strategies may be employed to block glutaminolysis. GLS1 is an especially attractive target given the emergence of several small molecule allosteric inhibitors [121]. Most promising is CB-839, an orally available drug and a potent GLS1 inhibitor that has been proven safe and efficacious in blocking tumor growth in preclinical studies and is currently undergoing clinical trials against a battery of cancers [122]. Aside from targeting GLS1, the directing of Glu to the Krebs cycle by Glu dehydrogenase or amino acid transaminase should be considered. Pharmacological inhibition of Glu dehydrogenase by epigallocatechin gallate and R162 or amino acid transaminase by aminooxyacetate inhibits tumor growth by disrupting the anaplerotic use of Gln in the Krebs cycle and may prove useful in vascular hyperproliferative disorders [123,124]. Alternatively, the blockade of GS by l-methionine sulfoximine inhibits angiogenesis in animal models of blinding eye and psoriatic skin diseases, meriting further investigation of GS targeting in pathological angiogenesis [51]. Thus, the metabolism of Gln offers multiple unique opportunities for therapeutic intervention. 

## 8. Conclusions and Perspectives

Amino acid therapy for cardiovascular disease represents a promising area of investigation and clinical application. While most of the research has focused on l-arginine, evolving studies have implicated a decrease in Gln bioavailability and an increase in glutaminolysis with the development of cardiovascular disease. A decrease in circulating Gln is observed in patients with cardiometabolic and sickle cell disease, which are improved by enteral administration of Gln. However, more detailed pharmacokinetic studies are needed to establish optimal dosage regimens in these disorders. In addition, Gln supplementation must be carefully evaluated in patients with elevated glutaminolysis as Gln may be directed towards harmful metabolic pathways that worsen cardiovascular health. In these instances, pharmacological targeting of glutaminolytic enzymes provides a promising strategy in mitigating cardiovascular disease. Future translational studies utilizing Gln delivery approaches and/or glutaminolysis inhibitors will ultimately determine the success of targeting Gln in cardiovascular disease.

## References

[1] Benjamin E.J., Blaha M.J., Chiuve S.E., Cushman M., Das S.R., Deo R., de Ferranti S.D., Floyd J., Fornage M., Gillespie C. (2017). American Heart Association Statistics Committee and Stroke Statistics Subcommittee. Heart disease and stroke statistics-2017 update. Circulation.

[2] Tarride J.E., Lim M., Desmeules M., Luo W., Burke N., O’Reilly D., Bowen J., Goeree R. (2009). A review of the cost of cardiovascular disease. Can. J. Cardiol..

[3] Wu G., Morris S.M. (1998). Arginine metabolism: nitric oxide and beyond. Biochem. J..

[4] Durante W. (2001). Regulation of l-arginine transport and metabolism in vascular smooth muscle cells. Cell Biophys..

[5] Forstermann U., Sessa W.C. (2012). Nitric oxide synthase: regulation and function. Eur. Heart J..

[6] Kumar A., Palfrey H.A., Pathak R., Kadowitz P.J., Gettys T.W., Murthy S.N. (2017). The metabolism and significance of homocysteine in health and disease. Nutr. Metab..

[7] Song P., Ramprasath T., Wang H., Zou M.H. (2017). Abnormal kynurenine pathway of tryptophan catabolism in cardiovascular diseases. Cell. Mol. Life Sci..

[8] Bertero T., Perk D., Chan S.Y. (2019). The molecular rationale for therapeutic targeting of glutamine metabolism in pulmonary hypertension. Expert Opin. Ther. Targets.

[9] Xi P., Jiang Z., Zheng C., Lin Y., Wu G. (2011). Regulation of protein metabolism by glutamine: Implications for nutrition and health. Front. Biosci..

[10] DeBerardinis R.J., Cheng T. (2010). Q’s next: The diverse functions of glutamine in metabolism, cell biology, and cancer. Oncogene.

[11] Cruzat V., Rogero M.M., Keane K.N., Curi R., Newsholme P. (2018). Glutamine: metabolism and immune function, supplementation, and clinical translation. Nutrients.

[12] Bertero T., Oldham W.M., Cottrill K.A., Pisano S., Vanderpool R.R., Yu Q., Zhao J., Tai Y., Tang Y., Zhang Y.Y. (2016). Vascular stiffness mechanoactivates YAP/TAZ-dependent glutaminolysis to drive pulmonary hypertension. J. Clin. Invest..

[13] Guo Y.Y., Deng Y.J., Li X.Q., Ning Y., Lin X.P., Guo S.M., Chen M.X., Han M. (2016). Glutaminolysis was induced by TGF-β1 through PP2Ac regulated Raf-MEK-ERK signaling in endothelial cells. PLoS ONE.

[14] Nelson D., Rumsey W.L., Erecinska M. (1992). Glutamine catabolism by heart muscle: Properties of phosphate-activated glutaminase. Biochem. J..

[15] Boelens P.G., van Leeuwen P.A., Dejong C.H., Deutz N.E. (2005). Intestinal renal metabolism of l-citrulline and l-arginine following enteral or parenteral infusion of l-alanyl-L[2,15N]glutamine or l-[2,15N]glutamine in mice. Am. J. Physiol. Gastrointest. Liver Physiol..

[16] Ford E.S., Giles W.H., Dietz W.H. (2002). Prevalence of the metabolic syndrome among US adults: Findings from the Third National Health and Nutrition Examination Survey. Obstet. Gynecol. Surv..

[17] McNeill A.M., Rosamond W.D., Girman C.J., Golden S.H., Schmidt M.I., East H.E., Ballantyne C.M., Heiss G. (2005). The metabolic syndrome and 11-year risk of incident cardiovascular disease in the atherosclerosis risk in communities study. Diabetes Care.

[18] Ford E.S. (2005). Risks for all-cause mortality, cardiovascular disease, and diabetes associated with the metabolic syndrome. Diabetes Care.

[19] Cheng S., Rhee E.P., Larson M.G., Lewis G.D., McCabe E.L., Shen D., Palma M.J., Roberts L.D., Dejam A., Souza A.L. (2012). Metabolite profiling identifies pathways associated with metabolic risk in humans. Circulation.

[20] Guasch-Ferre M., Hruby A., Toledo E., Clish C.B., Martinez-Gonzalez M.A., Salas-Salvado J., Hu F.B. (2016). Metabolomics in prediabetes and diabetes: A systemic review and meta-analysis. Diabetes Care.

[21] Newgard C.B., An J., Bain J.R., Muehlbauer M.J., Stevens R.D., Lien L.F., Haqq A.M., Shah S.H., Arlotto M., Slentz C.A. (2009). A branched-chain amino acid-related metabolic signature that differentiates obese and lean humans and contributes to insulin resistance. Cell Metab..

[22] Würtz P., Mäkinen V.P., Soininen P., Kangas A.J., Tukiainen T., Kettunen J., Savolainen M.J., Tammelin T., Viikari J.S., Rönnemaa T. (2012). Metabolic signatures of insulin resistance in 7,098 young adults. Diabetes.

[23] Wijekoon E.P., Skinner C., Brosnan M.E., Brosnan J.T. (2004). Amino acid metabolism in the Zucker fatty rat: Effects of insulin resistance and type 2 diabetes. Can. J. Physiol. Pharmacol..

[24] Wang S., Yang R., Wang M., Ji F., Li H., Tang Y., Chen W., Dong J. (2018). Identification of serum metabolites associated with obesity and traditional risk factors for metabolic disease in Chinese adults. Nutr. Metab. Cardiovasc. Dis..

[25] Wang S., Yu X., Zhang W., Ji F., Wang M., Yang R., Li H., Chen W., Dong J. (2018). Association of serum metabolites with impaired fasting glucose/diabetes and traditional risk factors for metabolic disease in Chinese adults. Clin. Chim. Acta.

[26] Ntzouvani A., Nomikos T., Panagiotakos D., Fragopoulou E., Pitsavos C., McCann A., Ueland P., Antonopoulou S. (2017). Amino acid profile and metabolic syndrome in a male Mediterranean population: A cross-sectional study. Nutr. Metab. Cardiovasc. Dis..

[27] Vangipurapu J., Stancáková A., Smith U., Kuusisto J., Laakso M. (2019). Nine amino acids are associated with decreased insulin secretion and elevated glucose levels in a 7.4-year follow-up study of 5,181 Finnish men. Diabetes.

[28] Greenfield J.R., Farooqi I.S., Keogh J.M., Henning E., Habib A.M., Blackwood A., Reimann F., Holst J.J., Gribble F.M. (2009). Oral glutamine increases circulating glucagon-like peptide 1, glucagon, and insulin concentrations in lean, obese, type 2 diabetic patients. Am. J. Clin. Nutr..

[29] Mansour A., Tehrani M.R.M., Qorbani M., Heshmat R., Larijani B., Hosseini S. (2015). Effect of glutamine supplementation on cardiovascular risk factors in patients with type 2 diabetes. Nutrition.

[30] Torres-Santiago L., Mauras N., Hossain J., Weltman A.L., Darmaun D. (2017). Does oral glutamine improve insulin sensitivity in adolescents with type 1 diabetes?. Nutrition.

[31] Hissa M.N., De Vasconcelos R.C., Guimarães S.B., Silva R.P., Garcia J.H.P., De Vasconcelos P.R.L. (2011). Preoperative glutamine infusion improves glycemia in heart surgery patients. Acta Cir. Bras..

[32] Bakalar B., Duska F., Pachl J., Fric M., Otahal M., Pazout J., Andel M. (2006). Parenterally administered dipeptide alanyl-glutamine prevents worsening of insulin sensitivity in multiple-trauma patients. Crit. Care Med..

[33] Iwashita S., Williams P., Jabbour K., Ueda T., Kobayashi H., Baier S., Flakoll P.J. (2005). Impact of glutamine supplementation on glucose homeostasis during and after exercise. J. Appl. Physiol..

[34] Petro A., Tevrizian A., Feinglos M.N., Surwit R.S., Opara E.C. (1996). L-Glutamine supplementation of a high fat diet reduces body weight and attenuates hyperglycemia and hyperinsulinemia in C57BL/6J mice. J. Nutr..

[35] Prada P.O., Hirabara S.M., De Souza C.T., Schenka A.A., Zecchin H.G., Vassallo J., Velloso L.A., Carneiro E., Carvalheira J.B.C., Curi R. (2007). RETRACTED ARTICLE: l-glutamine supplementation induces insulin resistance in adipose tissue and improves insulin signalling in liver and muscle of rats with diet-induced obesity. Diabetologia.

[36] Li C., Buettger C., Kwagh J., Matter A., Daikhin Y., Nissim I.B., Collins H.W., Yudkoff M., Stanley C.A., Matschinsky F.M. (2004). A signaling role for glutamine in insulin secretion. J. Biol. Chem..

[37] Stamler J., Brown I.J., Daviglus M.L., Chan Q., Kesteloot H., Ueshima H., Zhao L., Elliot P. (2009). For the INTERMAP Research Group. Glutamic acid, the main dietary amino acid, and blood pressure: The INTERMAP study (International Collaboration Study of Macronutrients, Micronutrients, and Blood Pressure). Circulation.

[38] Cabrera O., Jacques-Silva M.C., Speier S., Yang S.-N., Köhler M., Fachado A., Vieira E., Zierath J.R., Kibbey R., Berman D.M. (2008). Glutamate is a positive autocrine signal for glucagon release. Cell Metab..

[39] Cynober L.A. (1999). The use of alpha-ketoglutarate salts in clinical nutrition and metabolic care. Curr. Opin. Clin. Nutr. Metab. Care.

[40] Karaca M., Martin-Levilain J., Grimaldi M., Li L., Dizin E., Emre Y., Maechler P. (2018). Liver glutamate dehydrogenase controls whole body-body energy partitioning through amino acid-derived gluconeogenesis and ammonia homeostasis. Diabetes.

[41] Zheng Y., Hu F.B., Ruiz-Canela M., Clish C.B., Dennis C., Salas-Salvado J., Hruby A., Liang L., Toledo E., Corella D. (2016). Metabolites of glutamate metabolism are associated with incident cardiovascular events in the PREDIMED PREvencion con DIeta MEDiterranea PREDIMED) trial. J. Am. Heart Assoc..

[42] Qi L., Qi Q., Prudente S., Mendonca C., Andreozzi F., Di Pietro N., Sturma M., Novelli V., Mannino G.C., Formoso G. (2013). Association between a genetic variant of related to glutamic acid metabolism and coronary heart disease in type 2 diabetes. JAMA.

[43] Khogali S.E., Pringle S.D., Weryk B.V., Rennie M.J. (2002). Is glutamine beneficial in ischemic heart disease?. Nutrition.

[44] Sufit A., Weitzel L.B., Hamiel C., Queensland K., Dauber I., Rooyackers O., Wischmeyer P.E. (2012). Pharmacologically dosed oral glutamine reduces myocardial injury in patients undergoing cardiac surgery: A randomized pilot feasibility trial. Enteral. Nutr..

[45] Chavez-Tostado M., Carrill-Llamas F., Martinez-Gutierrez P.E., Alvarado-Ramirez A., Lopez-Taylor J.G., Vasquez-Jiminez J.C., Fuentes-Orozco C., Rendón-Félix J., Irusteta-Jiménez L., Calil-Romero V.C. (2017). Oral glutamine reduces myocardial damage after coronary revascularization under cardiopulmonary bypass. A random clinical trial. Nutr. Hosp..

[46] Ma W., Heianza Y., Huang T., Wang T., Sun D., Zheng Y., Hu F.B., Rexrode K.M., Manson J., Qi L. (2018). Dietary glutamine, glutamate, and mortality: two large prospective studies in US men and women. Int. J. Epidemiol..

[47] Epstein F.H., Vane J.R., Anggärd E.E., Botting R.M. (1990). Regulatory functions of the vascular endothelium. N. Engl. J. Med..

[48] Peyton K.J., Liu X.M., Yu Y., Yates B., Behnammanesh G., Durante W. (2018). Glutaminase-1 stimulates the proliferation, migration, and survival of human endothelial cells. Biochem. Pharmacol..

[49] Kim B., Li J., Jang C., Arany Z. (2017). Glutamine fuels proliferation but not migration of endothelial cells. EMBO J..

[50] Huang H., Vandekeere S., Kalucka J., Bierhansl L., Zecchin A., Bruning U., Visnagri A., Yuldasheva N., Goveia J., Cruys B. (2017). Role of glutamine and interlinked asparagine metabolism in vessel formation. EMBO J..

[51] Eelen G., Dubois C., Cantelmo A.R., Goveia J., Brüning U., DeRan M., Jarugumilli G., van Rijssel J., Saladino G., Comitani F. (2018). Role of glutamine synthetase in angiogenesis beyond glutamine synthesis. Nature.

[52] Unterluggauer H., Mazurek S., Lener B., Hütter E., Eigenbrodt E., Zwerschke W., Jansen-Dürr P. (2008). Premature senescence of human endothelial cells induced by inhibition of glutaminase. Biogerontology.

[53] Zhang W., Li H., Ogando D.G., Li S., Feng M., Price F.W., Tennessen J.M., Bonanno J.A. (2017). Glutaminolysis is essential for energy production and ion transport in human corneal endothelium. EBioMedicine.

[54] Hinshaw D.B., Burger J.M. (1990). Protective effect of glutamine on endothelial cell ATP in oxidant injury. J. Surg. Res..

[55] Parolari A., Sala R., Antona C., Bussolati O., Alamanni F., Mezzadri P., Dall’Asta V., Gazzola G.C., Biglioli P. (1997). Hypertonicity induces injury to cultured human endothelium: attenuation by glutamine. Ann. Thorac. Surg..

[56] Sanchez E.L., Carroll P.A., Thalhofer A.B., Lagunoff M. (2015). Latent KSHV infected endothelial cells are glutamine addicted and require glutaminolysis for survival. PLoS Pathog..

[57] Safi S.Z., Batumalaie K., Mansor M., Chinna K., Kumar S., Karimian H., Qvist R., Ashraf M.A., Yan G.O. (2015). Glutamine treatment attenuates hyperglycemia-induced mitochondrial stress and apoptosis in umbilical vein endothelial cells. Clinics (Sao Paulo).

[58] Parfenova H., Basuroy S., Bhattacharya S., Tcheranova D., Qu Y., Regan R.F., Leffler C.W. (2006). Glutamate induces oxidative stress and apoptosis in cerebral vascular endothelial cells: Contributions of HO-1 and HO-2 to cytoprotection. Am. J. Physiol. Physiol..

[59] Hsu C.S., Chou S.Y., Liang S.J., Chang C.Y., Yeh S.L. (2006). Effect of physiologic levels of glutamine on ICAM-1 expression in endothelial cells activated by preeclamptic plasma. J. Reprod. Med..

[60] Hou Y.C., Hsu C.S., Yeh C.L., Chiu W.C., Pai M.H., Yeh S.L. (2005). Effects of glutamine on adhesion molecule expression and leukocyte transmigration in endothelial cells exposed to arsenic. J. Nutr. Biochem..

[61] Su S.T., Yeh C.L., Hou Y.C., Pai M.H., Yeh S.L. (2017). Dietary glutamine supplementation enhances endothelial progenitor cell mobilization in streptozotocin-induced diabetic mice subjected to limb ischemia. J. Nutr. Biochem..

[62] Pai M.H., Shih Y.M., Shih J.M., Yeh C.L. (2016). Glutamine administration modulates endothelial progenitor cell and lung injury in septic mice. Shock.

[63] Addabbo F., Chen Q., Patel D.P., Rabadi M., Ratliff B., Zhang F., Jasmin J.F., Wolin M., Lisanti M., Goligorsky M.S. (2013). Glutamine supplementation alleviates vasculopathy and corrects metabolic profile in an in vivo model of endothelial cell dysfunction. PLoS ONE.

[64] Ellis A.C., Patterson M., Dudenbostel T., Ccalhoun D., Gower B. (2016). Effects of 6-month supplementation with β-hydroxy-β-methybutyrate, glutamine and arginine on vascular endothelial function of older adults. Eur. J. Clin. Nutr..

[65] Hecker M., Mitchell J.A., Swierkosz T.A., Sessa W.C., Vane J.R. (1990). Inhibition by l-glutamine of the release of endothelium-derived relaxing factor from cultured endothelial cells. Br. J. Pharmacol..

[66] Meininger C.J., Wu G. (1997). L-glutamine inhibits nitric oxide synthesis in bovine venular endothelial cells. J. Pharmacol. Exp. Ther..

[67] Wu G., Haynes T.E., Li H., Yan W., Meininger C.J. (2001). Glutamine metabolism to glucosamine is necessary for glutamine inhibition of endothelial nitric oxide synthase. Biochem. J..

[68] Wu G., Haynes T.E., Yan W., Meininger C.J. (2001). Presence of glutamine:fructose-6-phosphate amidotransferase for glucosamine-6-phosphate synthesis in endothelial cells: Effect of hyperglycemia and glutamine. Diabetologia.

[69] Leighton B., Curi R., Hussein A., Newsholme E.A. (1987). Maximum activities of some key enzymes of glycolysis, glutaminolysis, Krebs cycle and fatty acid utilization in bovine pulmonary endothelial cells. FEBS Lett..

[70] Wu G., Haynes T.E., Li H., Meininger C.J. (2000). Glutamine metabolism in endothelial cells: Ornithine synthesis from glutamine via pyrroline-5-carboxylate synthase. Comp. Biochem. Physiol. Part A Mol. Integr. Physiol..

[71] Liu X.M., Peyton K.J., Durante W. (2017). Ammonia promotes endothelial cell survival via the heme oxygenase-1 mediated release of carbon monoxide. Free Radic. Biol. Med..

[72] Durante W. (2006). Role of carbon monoxide in cardiovascular function. J. Cell. Mol. Med..

[73] Durante W., Johnson F.K., Johnson R.A. (2010). Targeting heme oxygenase-1 in vascular disease. Curr. Drug Targets.

[74] Durante W. (2011). Protective role of heme oxygenase-1 against inflammation in atherosclerosis. Front. Biosci..

[75] Peyton K.J., Reyna S.V., Chapman G.B., Ensenat D., Liu X.M., Wang H., Schafer A.I., Durante W. (2002). Heme oxygenase-1-derived carbon monoxide is an autocrine inhibitor of vascular smooth muscle cell growth. Blood.

[76] Peyton K.J., Shebib A.R., Azam M.A., Liu X.-M., Tulis D.A., Durante W. (2012). Bilirubin inhibits neointima formation and vascular smooth muscle cell proliferation and migration. Front. Pharmacol..

[77] Eng C.H., Yu K., Lucas J., White E., Abraham R.T. (2010). Ammonia derived from glutaminolysis is a diffusible regulator of autophagy. Sci. Signal.

[78] Abusneina A., Gauthier E.R. (2016). Ammonium ions improve the survival of glutamine-starved hybridoma cells. Cell Biosci..

[79] Rabinovitch M. (2012). Molecular pathogenesis of pulmonary arterial hypertension. J. Clin. Invest..

[80] Chan Y., Loscalzo J. (2008). Pathogenic mechanisms of pulmonary arterial hypertension. J. Mol. Cell. Cardiol..

[81] Ge J., Cui H., Xie N., Banerjee S., Guo S., Dubey S., Barnes S., Liu G. (2018). Glutminolysis promotes collagen translation and stability via α-ketoglutarate-mediate mTOR activation and proline hydroxylation. Am. J. Respir. Cell Mol. Biol..

[82] Piao L., Fang Y.-H., Parikh K., Ryan J.J., Toth P.T., Archer S.L. (2013). Cardiac glutaminolysis: a maladaptive cancer metabolism pathway in the right ventricle in pulmonary hypertension. J. Mol. Med..

[83] Egnatchik R.A., Brittain E.L., Shah A., Farres W.H., Ford H.J., Monahan K., Kang C.J., Kocurek E.G., Zhu S., Luong T. (2017). Dysfunctional BMPR2 signaling drives an abnormal endothelial requirement for glutamine in pulmonary arterial hypertension. Pulm. Circ..

[84] Zabot G.P., Carvalhal G.F., Marroni N.P., Hartmann R.M., Da Silva V.D., Fillmann H.S. (2014). Glutamine prevents oxidative stress in a model of mesenteric ischemia and reperfusion. World J. Gastroenterol..

[85] Kim K.S., Suh G.J., Kwon W.Y., Lee H.J., Jeong K.Y., Jung S.K., Kwak Y.H. (2013). The effect of glutamine on cerebral ischaemic injury after cardiac arrest. Resuscitation.

[86] Shih Y.M., Shih J.M., Pai M.H., Hou Y.C., Yeh C.L., Yeh S.L. (2016). Glutamine administration after sublethal lower limb ischemia reduces inflammatory reaction and offers organ protection in ischemia/reperfusion injury. J. Parenter. Enter. Nutr..

[87] Prem J.T., Eppinger M., Lemmon G., Miller S., Nolan D., Peoples J. (1999). The role of glutamine in skeletal muscle ischemia/reperfusion injury in the rat hind limb model. Am. J. Surg..

[88] Zhang S.C., Shi Q., Feng Y.N., Fang J. (2013). Tissue protective effect of glutamine on hepatic ischemia-reperfusion injury via the induction of heme oxygenase-1. Pharmacology.

[89] Esposito E., Mondello S., Di Paola R., Mazzon E., Italiano D., Paterniti I., Mondello P., Aloisi C., Cuzzocrea S. (2011). Glutamine contributes to ameliorate inflammation after renal ischemia/reperfusion injury in rats. Naunyn Schmiedeberg’s Arch. Pharmacol..

[90] Bolotin G., Raman J., Williams U., Bacha E., Kocherginsky M., Jeevanandam V. (2007). Glutamine improves myocardial infarction following ischemia-reperfusion injury. Asian Cardiovasc. Thorac. Ann..

[91] Pai M.H., Lei C.S., Su S.T., Yeh S.L., Hou Y.C. (2019). Effects of dietary glutamine supplementation on immune cell polarization and muscle regeneration in diabetic mice with limb ischemia. Eur. J. Nutr..

[92] Stangl R., Szijártó A., Onody P., Tamas J., Tátrai M., Hegedüs V., Blázovics A., Lotz G., Kiss A., Módis K. (2011). Reduction of liver ischemia-reperfusion injury via glutamine pretreatment. J. Surg. Res..

[93] Luo L.L., Li Y.F., Shan H.M., Wang L.P., Yuan F., Ma Y.Y., Li W.L., He T.T., Wang Y.Y., Qu M.J. (2019). L-glutamine protects mouse brain from ischemic injury via up-regulating heat shock protein 70. CNS Neurosci. Ther..

[94] Zhang Y., Zou Z., Li Y., Yuan H., Shi X.Y., Shi X. (2009). Glutamine-induced heat shock protein protects against renal ischaemia-reperfusion injury in rats. Nephrology.

[95] Wang A.L., Niu Q., Shi N., Wang J., Jia X.F., Lian H.F., Liu Z., Liu C.X. (2015). Glutamine ameliorates intestinal ischemia-reperfusion Injury in rats by activating the Nrf2/Are signaling pathway. Int. J. Clin. Exp. Pathol..

[96] Korthuis R.J., Durante W. (2005). Heme oxygenase-1: A pluripotent sentinel limiting the systemic inflammatory response to extremity ischemia and reperfusion. Crit. Care Med..

[97] Morris C.R., Suh J.H., Hagar W., Larkin S., Bland D.A., Steinberg M.H., Vichinsky E.P., Shigenaga M., Ames B., Kuypers F.A. (2008). Erythrocyte glutamine depletion, altered redox environment, and pulmonary hypertension in sickle cell disease. Blood.

[98] Niihara Y., Matsui N.M., Shen Y.M., Akiyama D.A., Johnson C.S., Sunga M.A., Magpayo J., Embury S.H., Kalra V.K., Cho S.H. (2005). L-Glutamine therapy reduces endothelial adhesion of sickle red blood cells to human umbilical vein endothelial cells. BMC Blood Disord..

[99] Niihara Y., Miller S.T., Kanter J., Lanzkron S., Smith W.R., Hsu L.L., Gordeuk V.R., Viswanathan K., Sarnaik S., Osunkwo I. (2018). A phase 3 clinical trial of l-glutamine in sickle cell disease. N. Engl. J. Med..

[100] Cao Y., Kennedy R., Klimberg V. (1999). Glutamine protects against doxorubicin-induced cardiotoxicity. J. Surg. Res..

[101] Todorova V., Vanderpool D., Blossom S., Nwokedi E., Hennings L., Mrak R., Klimberg V.S. (2009). Oral glutamine protects against cyclophosphamide-induced cardiotoxicity in experimental rats through increase of cardiac glutathione. Nutrition.

[102] Yan H., Zhang Y., Lv S.J., Wang L., Liang G.P., Wan Q.X., Peng X. (2012). Effects of glutamine treatment on myocardial damage and cardiac function in rats after severe burn injury. Int. J. Clin. Exp. Pathol..

[103] Badole S.L., Jangam G.B., Chaudhari S.M., Ghule A.E., Zanwar A.A. (2014). L-Glutamine supplementation prevents the development of experimental diabetic cardiomyopathy in streptozotocin-nicotinamide induced diabetic rats. PLoS ONE.

[104] Shao M., Huang C., Li Z., Yang H., Feng Q. (2015). Effects of glutamine and valsartan on the brain natriuretic peptide and N-terminal pro-B-type natriuretic peptide of patients with chronic heart failure. Pak. J. Med. Sci..

[105] Wu C., Kato T.S., Ji R., Zizola C., Brunjes D.L., Deng Y., Akashi H., Armstrong H.F., Kennel P.J., Thomas T. (2015). Supplementation of l-alanyl-l-glutamine and fish oil improves body composition and quality of life in patients with chronic heart failure. Circ. Hear. Fail..

[106] Hankard R.G., Darmaun D., Sager B.K., D’Amore D., Parsons W.R., Haymond M. (1995). Response of glutamine metabolism to exogenous glutamine in humans. Am. J. Physiol. Metab..

[107] Matthews D.E., Marano M.A., Campbell R.G. (1993). Splanchnic bed utilization of glutamine and glutamic acid in humans. Am. J. Physiol. Metab..

[108] Ziegler T.R., Benfell K., Smith R.J., Young L.S., Brown E., Ferrari-Baliviera E., Lowe D.K., Wilmore D.W. (1990). Safety and metabolic effects of l-glutamine administration in Humans. J. Parenter. Enter. Nutr..

[109] Klassen P., Mazariegos M., Solomons N.W., Furst P. (2000). The pharmacokinetic responses of humans to 20 g of alanyl-glutamine dipeptide differ with the dosing protocol but not with gastric acidity or in patients with acute dengue fever. J. Nutr..

[110] Cruzat V.F., Rogero M.M., Tirapegui J. (2010). Effects of supplementation with free glutamine and the dipeptide alanyl-glutamine on parameters of muscle damage and inflammation in rats submitted to prolonged exercise. Cell Biochem. Funct..

[111] Adibi S.A. (2003). Regulation of expression of the intestinal oligopeptide transporter (Pept-1) in health and disease. Am. J. Physiol. Gastrointest. Liver Physiol..

[112] Morris C.R., Hamilton-Reeves J., Martindale R.G., Sarav M., Gautier J.B.O. (2017). Acquired amino acid deficiencies: a focus on arginine and glutamine. Nutr. Clin. Pract..

[113] Johnson F.K., Peyton K.J., Liu X.M., Azam M.A., Shebib A.R., Johnson R.A., Durante W. (2015). Arginase promotes endothelial dysfunction and hypertension in obesity. Obesity.

[114] Tang W.H.W., Wang Z., Cho L., Brennan D.M., Hazen S.L. (2009). Diminished global arginine bioavailability and increased arginine catabolism as metabolic profile of increased cardiovascular risk. J. Am. Coll. Cardiol..

[115] Heyland D., Muscedere J., Wischmeyer P.E., Cook D., Jones G., Albert M., Elke G., Berger M.M., Day A.G. (2013). A randomized trial of glutamine and antioxidants in critical ill patients. N. Engl. J. Med..

[116] Van Zanten A.R., Sztark F., Kaisers U.X., Zielmann S., Felbinger T.W., Sablotzki A.R., De Waele J.J., Timsit J.F., Honing M.L., Keh D. (2014). High-protein enteral nutrition enriched with immune-modulating nutrients vs standard high-protein enteral nutrition and nosocomial infections in the ICU: A randomized clinical trial. JAMA.

[117] Motterlini R., Otterbein L.E. (2010). The therapeutic potential of carbon monoxide. Nat. Rev. Drug Discov..

[118] Kim H.-H., Choi S. (2018). Therapeutic aspects of carbon monoxide in cardiovascular disease. Int. J. Mol. Sci..

[119] Yu X.H., Cui L.B., Wu K., Zheng X.L., Cayabyab F.S., Chen Z.W., Tang C.K. (2014). Hydrogen sulfide as a potent cardiovascular protective agent. Clin. Chim. Acta.

[120] Zhang L., Wang Y., Li Y., Li L., Xu S., Feng X., Liu S. (2018). Hydrogen sulfide (H2S)-releasing compounds: therapeutic potential in cardiovascular diseases. Front. Pharmacol..

[121] Zimmerman S.C., Duvall B., Tsukamoto T. (2019). Recent progress in the discovery of allosteric inhibitors of kidney type glutaminase. J. Med. Chem..

[122] Gross M.I., Demo S.D., Dennison J.B., Chen L., Chernov-Rogan T., Goyal B., Janes J.R., Laidig G.J., Lewis E.R., Li J. (2014). Antitumor activity of the glutaminase inhibitor CB-839 in triple-negative breast cancer. Mol. Cancer Ther..

[123] Choi Y.K., Park K.G. (2018). Targeting glutamine metabolism for cancer treatment. Biomol. Ther..

[124] Korangath P., Teo W.W., Sadik H., Han L., Mori N., Huijts C.M., Wildes F., Bharti S., Zhang Z., Santa-Maria C.A. (2015). Targeting glutamine metabolism in breast cancer with aminooxyacetate. Clin. Cancer Res..

